# Methodological guidelines for Health Technology Assessment in Oman

**DOI:** 10.1080/20523211.2025.2596523

**Published:** 2025-12-09

**Authors:** Said Wani, Hilal Alsabti, Said Allamki, Ahmed Almandhari, Suleiman Alhajji, Ibrahim Alrashdi, Raid Al Sabri, Adnan Alqassabi, Ahmed Alzaabi, Aisha Almaashani, Alia Ashuaili, Anisa Rasool, Asiya Alkindi, Bushra Salman, Faiza Alzadjali, Khalid Al Rahbi, Khulood Almutawa, Manal Alansari, Mardheya AlKharusi, Nadiya AlBulushi, Rahma Alghadani, Rana Aljaber, Safiya Almuaini, Said Alkindi, Salim Alzawamri, Sara Albalushi, Suha Al lawati, Zuhair Alsalti, Hassan AlBalushi, Ahmad Nader Fasseeh, Zoltán Kaló

**Affiliations:** a Center for Health Technology Assessment, Semmelweis University, Budapest, Hungary; b Center for Pharmacology and Drug Research & Development, Semmelweis University, Budapest, Hungary; c Ministry of Health, Muscat, Oman; d Minister of Health, Ministry of Health, Muscat, Oman; e Royal Hospital, Ministry of Health, Muscat, Oman; f Ministry of Health, Sohar, Oman; g Sultan Qaboos Hospital, Ministry of Health, Salalah, Oman; h Pharmacy Department, National Hematology and Bone Marrow Transplant Center, University Medical City, Seeb, Oman; i Drug Safety Center, Ministry of Health, Muscat, Oman; j Sumail Hospital, Ministry of Health, Sumail, Oman; k Khoula Hospital, Muscat, Oman; l Al Nahdha Hospital, Ministry of Health, Muscat, Oman; m Pharmaceutical Care Department, Ministry of Health, Muscat, Oman; n Ministry of Health, Rustaq, Oman; o Sultan Qaboos Comprehensive Cancer Care and Research Centre, University Medical City, Seeb, Oman; p Khoula Hospital, Ministry of Health, Muscat, Oman; q Ministry of Health, Salalah, Oman; r Ibri Hospital, Ministry of Health, Ibri, Oman; s Syreon Middle East, Alexandria, Egypt; t Faculty of Pharmacy, Alexandria University, Alexandria, Egypt; u Syreon Research Institute, Budapest, Hungary

**Keywords:** Health technology assessment, value judgment, evidence based health policy, guidelines, transparency, Oman

## Abstract

**Background:**

Health Technology Assessment (HTA) supports optimal healthcare resource allocation by providing evidence-based input for decision-making. The manuscript aims to: (1) summarise the development process of methodological guidelines for Oman with involvement of key stakeholders to facilitate consistency, transparency and robustness in technology appraisal, and (2) present the features of the accepted HTA guidelines.

**Methods:**

Guideline development included five steps: (1) learnings from international HTA guidelines; (2) multistakeholder workshop on key components of the Omani methodological guidelines; (3) survey to facilitate consensus on most critical elements; (4) drafting the guidelines based on collected observations; and (5) validation through feedback from policy-makers and HTA experts.

**Results:**

The Omani HTA guidelines consist of six sections: target indication, medical assessment, economic evaluation, budget impact analysis, social and ethical considerations, and transparency. Health benefit evaluation should prioritise policy-relevant outcomes, including mortality, morbidity, and quality of life. Using clinical assessment reports published by reputable HTA agencies is advocated. When added value is demonstrated over a policy-relevant comparator, cost-utility analysis with QALYs calculation, a 3% discount rate, and cost-effectiveness thresholds is the preferred approach. Budget impact analysis should cover a four-year horizon. Full disclosure of conflicts of interest, expert contributions, and public availability of HTA dossiers are mandatory to enhance transparency.

**Conclusion:**

Acceptance of Omani HTA methodological guidelines by the Ministry of Health represent an important milestone in improving the evidence base of policy decisions in Oman. The guidelines offer a standardised, transparent framework to judge the value and affordability of health technologies in alignment with international standards and local healthcare needs and priorities.

## Background

Health technology assessment plays a vital role in judging the value of health technologies and informing decision-makers about how to optimise resource allocation in the healthcare system in order to maximise health gain from scarce resources (Mitton et al., 2019).

To integrate the value judgment in healthcare policies, it is important to align methodologies and processes with local decision-making contexts. In recent years, the Sultanate of Oman has made important steps to implement its HTA system according to local needs. Iterative workshops and a comprehensive training programme were organised for multiple local stakeholders to increase limited HTA capacities. An HTA roadmap with actionable items was developed by key policy-makers (Al Rashdi et al., 2024). The published HTA roadmap highlighted the need to ensure the quality of HTA reports by preparing local methodological guidelines for HTA.

HTA guidelines must be comprehensive, standardised, and contextually tailored to the specific needs and challenges of the country (Daccache et al., 2022; Eddama & Coast, 2008). A standardised methodological framework ensures that technology appraisals are carried out consistently across different technologies and promotes transparency in the process through clear documentation of methodologies, assumptions, and decision-making criteria (Al Rashdi et al., 2024; Kaló et al., 2013).

The manuscript aims (1) to summarise the process of developing methodological guidelines for Oman with the involvement of key stakeholders to facilitate consistency, transparency, and robustness in technology appraisal, and (2) to present the key features of the accepted HTA guidelines. Although the cost-effectiveness threshold (CET) framework is included in the HTA methodological guidelines, it is detailed in a separate standalone manuscript (Wani et al., 2025).

## Methods

Five steps were taken to develop the Omani HTA methodological guidelines, including (1) learning from international HTA guidelines; (2) multistakeholder workshop on key components of the Omani methodological guidelines; (3) survey to facilitate consensus on the most critical elements; (4) drafting of guidelines based on collected observations; and (5) validation through feedback from policy-makers and HTA experts.

### Learnings from international guidelines

Interestingly, a review of publications from the scientific literature does not result in complete information about methodological guidelines in different countries, as there is no guarantee that the guideline developers make a scientific publication when they develop or update the methodological guidelines. Therefore, the targeted review, which was completed independently from the Omani HTA guideline development initiative, focused on guidelines presented on the ISPOR website (Korra et al., 2024). The learnings from this targeted review provided the foundation for the key features and main chapters of the HTA guidelines in Oman.

### Multistakeholder workshop

A multistakeholder workshop was organised in Muscat in March 2024 for multiple stakeholders, including regulators, payers, policy-makers, and healthcare providers. 25 participants represented the Ministry of Health and University Medical City. The workshop aimed to facilitate consensus on the key components and most sensitive issues of the methodological guidelines. After presenting an overview of international approaches based on conclusions from the targeted literature review, participants engaged in an open discussion about key components and chapters of the methodological guidelines. Participants agreed that consensus on the most sensitive issues in the methodological guidelines would be facilitated by an anonymous survey.

### Multistakeholder survey

The participants in the multistakeholder workshop were asked to express their opinions in an anonymous survey by using Mentimeter® about topics identified in the previous step. The survey followed a flowchart style, where new questions popped up based on previous answers to keep the voting relevant and organised. This enabled the questions to stay relevant and the vote to progress through the remaining rounds.

### Guideline preparation

Based on the conclusions of the multistakeholder workshop, a draft version of the HTA guidelines was formed in six sections, including target indications, medical assessment, economic evaluation, budget impact analysis, social and ethical considerations, and transparency.

### Guideline validation

The draft version of the guideline was sent for review by email to those stakeholders and experts who had contributed to the workshop. Building on the stakeholders’ feedback the recommendations were amended. Stakeholders and experts reached consensus on the basic structure and core elements of the guideline.

## Results

### Learnings from international guidelines

Most of the 54 guidelines published on the ISPOR website came from developed countries with established HTA agencies (Korra et al., 2024). Key components of the international HTA guidelines included PICO framework (i.e. targeted patients, intervention, comparator, outcomes), clinical assessment, economic evaluation, budget impact analysis, other aspects and transparency.

The most impactful issues in international guidelines were the preferred method of calculating health gain, transferability of international data, explicit threshold values, discount rates, and transparency of HTA reports. It was interesting to observe that the methodological guidelines of smaller countries did not specify any unique methodologies that are not used in many other countries, and they recommended the transferability of evidence on relative effectiveness. The majority of guidelines recommended the use of cost-utility analysis (CUA) for economic evaluation with quality-adjusted life years (QALYs) as the preferred measure of health gain. An adequate – potentially lifelong – time horizon was recommended in the guidelines to explore future costs and benefits that arise from the technology invested. Discount rates were typically set between 3% and 5% for calculating the present value of future benefits and costs used to assess effects in the long term. Typically, the most common comparator recommended is the standard of care with public reimbursement (Korra et al., 2024).

### Multistakeholder workshop

After reviewing key learnings from international methodological guidelines, participants in the multistakeholder workshop had a guided discussion on why to have local methodological guidelines. They agreed that a standardised HTA assessment process through clear methodological guidelines has the potential to enhance consistency and reliability in assessments. Participants also had consensus about the main chapters of the guidelines, including target indication, medical assessment, economic evaluation, budget impact analysis, social and ethical considerations, and transparency (including publishing HTA reports and declaration of conflict of interest). Finally, they also concluded on the most sensitive topics, which required anonymous voting.

### Multistakeholder survey

Out of 25 survey respondents, 24 participants voted for creating local HTA guidelines specifically tailored to the needs and priorities of Oman's healthcare system with the addition of regular updates. Furthermore, 92% of participants expressed support for introducing a checklist for critical appraisal with a mandatory self-appraisal by pharmaceutical and medical device manufacturers to enhance the standardisation of both technology assessment and appraisal practices.

While QALYs are not perfect measures for quantifying the aggregated health gain over the long term, pharmaceutical and medical device companies traditionally have estimates of the QALY gain, which can be used for policy decisions in case of explicit threshold values. Therefore, all participants supported the use of QALYs in the base case analysis.

During the multistakeholder workshop, participants reached consensus on using the same discount rates for future health outcomes and costs. The voting results for the discount rates were equal at 2.5%, 3%, and 3.5%, and the median value was calculated. Survey results showed a high level of agreement on the adequate acceptability of international data, with 78% in favour of using international epidemiological data if local data does not exist. 87% advocated the use of international quality of life data, and 96% agreed with the transferability of international safety and effectiveness data. However, the use of international cost data was rejected, indicating a preference for using local context-specific economic inputs.

The support for transparency in HTA process was also voted upon, with the majority of participants agreeing that publication of key components of HTA reports should be mandated or at least recommended (see Table 1). However, 52% of participants agreed that the proposed price of the new technology should not be in the public domain, as confidentiality provides more room for local payers to negotiate pharmaceutical prices.

**Table 1.** Results of the multistakeholder survey.

Questions	Options	Votes (*n*)	Votes (%)
**Part 1: Standardisation and localisation of technology assessment and critical appraisal methods**			
Development of local HTA methodological guidelines	Not needed, only international guidelines should be applied	1	4
	Local methodological guideline should be published	0	0
	Local methodological guideline should be published & revised every 3 years	24	96
Use of critical appraisal checklist	No need for critical appraisal checklist, only international standards should be applied	0	0
	Critical appraisal checklist developed & used by the HTA Agency, but not published	1	4
	Critical appraisal checklist developed & used by the HTA Agency and published	1	4
	Critical appraisal checklist developed & used by the HTA Agency and published; self-appraisal part of submitted HTA dossier	22	92
**Part 2: Critical methodological questions**^a^			
The prefered methodology for calculating health gain should be	Quality adjusted life years (QALYs)	22	100
	Clinical endpoints	0	0
	Other measures	0	0
Discount rate	1.5%	0	0
	2.0%	0	0
	2.5%	7	33
	3.0%	7	33
	3.5%	7	33
	4.0%	0	0
	4.5%	0	0
	5.0%	0	0
**Part 3: Acceptability of international data?**			
Epidemiology of the target indication (incidence, prevalence)	Acceptable	18	78
	Not acceptable	5	22
Relative effectiveness of the new technology	Acceptable	21	91
	Not acceptable	2	9
Safety of the new technology	Acceptable	22	96
	Not acceptable	1	4
Quality of life of patients in different health states	Acceptable	20	87
	Not acceptable	3	13
Resource use data	Acceptable	8	35
	Not acceptable	15	65
Cost data (e.g. unit cost of health care resources)	Acceptable	0	0
	Not acceptable	23	100
**Part 4: Transparency of HTA dossiers submitted by pharmaceutical and medical device companies – which elements should be accessible on a public website for patients, clinicians and researchers?**			
Epidemiology of the target indication (incidence, prevalence)	Publication is mandatory	15	65
	Publication is recommended	7	30
	No publication	1	4
Current patient pathways with highlights on unmet medical need	Publication is mandatory	13	57
	Publication is recommended	8	35
	No publication	1	4
Efficacy and safety of the new technology	Publication is mandatory	17	74
	Publication is recommended	3	13
	No publication	3	13
Methodology of calculating the health gain by the new technology	Publication is mandatory	10	43
	Publication is recommended	11	48
	No publication	2	9
Estimated health gain	Publication is mandatory	14	61
	Publication is recommended	6	26
	No publication	3	13
Methodology of cost calculations	Publication is mandatory	9	39
	Publication is recommended	10	43
	No publication	4	17
Estimated current resource use and treatment costs of patients	Publication is mandatory	7	30
	Publication is recommended	11	48
	No publication	5	22
Proposed price of the new technology	Publication is mandatory	7	30
	Publication is recommended	4	17
	No publication	12	52
Estimated resource use and treatment costs of patients with the new technology	Publication is mandatory	6	26
	Publication is recommended	8	35
	No publication	9	39
Economic modelling methodology (model type, time horizon, discount rate, etc.)	Publication is mandatory	7	30
	Publication is recommended	9	39
	No publication	7	30
Cost-effectiveness results (incr. health gain, costs and cost-effectiveness ratio)	Publication is mandatory	12	52
	Publication is recommended	8	35
	No publication	3	13
Sensitivity analysis results for the cost-effectiveness analysis	Publication is mandatory	7	30
	Publication is recommended	10	43
	No publication	6	26
Methodology of budget impact calculations	Publication is mandatory	11	48
	Publication is recommended	8	35
	No publication	4	17
Current treatment mix of patients	Publication is mandatory	11	48
	Publication is recommended	11	48
	No publication	1	4
Estimated patient numbers and market share of new technology in next 3–5 years	Publication is mandatory	7	30
	Publication is recommended	12	52
	No publication	4	17
Budget impact of the new technology	Publication is mandatory	10	43
	Publication is recommended	8	35
	No publication	5	22

^a^ Survey items related to cost-effectiveness thresholds are reported in a separate manuscript.

### Features of the HTA methodological guidelines

The most important features of the Omani HTA methodological guidelines are summarised below.

#### Target indication

The target indication, target population, and the number of patients within the target population seeking reimbursement must be clearly defined. The target indication for reimbursement may be more specific but not broader than the registered indication. The population in the HTA dossier cannot differ from the targeted patient population in the reimbursement application. When the population in the HTA dossier is narrower than the population in the reimbursement application, it is essential to evaluate the generalizability of the conclusions.

#### Medical assessment

The disease area has to be detailed in four chapters. When local epidemiological data are limited, international incidence and prevalence figures are acceptable with a preference for data from Gulf countries or the Middle East, especially if they are published either by the World Health Organization (WHO) or the Global Burden of Disease initiative or reputable academic institutions. Current management of the condition should cover information on the current standard diagnosis, treatment, and care practices for the specified indication, where possible supported by published local and international guidelines. Description of patient pathways for the disease should specify the settings in which the care is provided (e.g. primary care, outpatient and inpatient specialist care, home care). Unmet needs that are not fully addressed by standard technologies or the standard of care (i.e. the best available treatment or practice, supported by clinical evidence and widely used as the current routine practice) should be clearly articulated, such as late detection, low cure rates, therapy resistance, adherence issues, severe side effects, etc. Finally, an overview of current reimbursed technologies in the target indication should be provided.

The choice of the comparator health technology has to be justified. In reimbursement submissions, the recommended comparator is a reimbursed health technology that is included in national and international clinical practice guidelines and may be substituted by the investigated health technology. An overview of the effectiveness and safety of the comparator health technology is necessary.

A comprehensive review of the investigated health technology includes its therapeutic indications, overview on scientific evidence from clinical trials, real-world studies, and the estimated health gain. If several randomised clinical trials are accessible, meta-analysis or network meta-analysis is recommended to aggregate their results. It is important to give the sources of clinical data and the details of the calculations in a detailed, transparent, and reproducible manner. Indirect evidence may also be considered in the health technology assessment. In assessing the health gain anticipated from the utilisation of the health technology, the evaluation of health benefits should primarily focus on policy-relevant outcomes, such as mortality, morbidity, and health-related quality of life. Utilisation of clinical assessment reports published by reputable HTA agencies (e.g. Joint Clinical Assessment reports prepared according to the European HTA regulation) is advocated as the primary source of evidence on the health gain.

#### Economic evaluation

The Omani HTA methodological guidelines present the economic evaluation section in a comprehensively detailed manner. All relevant costs should be presented in local currency Omani Riyal (OMR) and considered from the perspective of the healthcare system. The primary analysis should focus on direct healthcare costs, however, supplementary analyses may consider societal perspective by including costs like productivity losses and caregiver burden.

QALYs take into account all benefits and adverse events of the technology in the combination of patient survival and health related quality of life and so they are the preferred measures in reporting health outcomes. If the investigational technology demonstrates a clinically meaningful and statistically significant improvement in the primary endpoints of clinical trials, CUA should be applied to judge its economic value. When no meaningful and statistically significant health gain is demonstrated in clinical trials, cost-minimization analysis can be used.

The time horizon used in the model should be long enough to accurately reflect the costs and outcomes associated with the investigated health technology, ensuring that both short-term and long-term consequences are fully considered. A lifetime horizon is recommended for chronic diseases or when treatment impacts mortality rates. For acute illnesses, a shorter time horizon may be appropriate, but this should be clearly justified. An inadequate time horizon can bias the analysis and misrepresent the long-term cost-effectiveness of an intervention. Scenario analysis is also encouraged to assess the robustness of results across varying time horizons.

The economic evaluation of health technologies involves using models, such as decision trees, Markov models, and simulations to estimate long-term costs and outcomes. Key recommendations include clearly defining the study question, justifying the model's scope and structure, ensuring transparent input sources, and aligning the model with the research objectives and health technologies under review.

The future costs and outcomes are recommended to be discounted at 3% per annum to reflect time preference and ensure comparability of present values. Presenting the results should be in a clear and transparent way by using the incremental cost-effectiveness ratio (ICER). The calculated ICER should be displayed clearly in a tabular format for improved comparability across HTA reports (see Table 2).

**Table 2.** Template for reporting results of the economic evaluation.

	Cost	Expected health effect	Incremental cost	Incremental health	ICER
**Health benefit expressed in life years**					
Investigated health technology	XXX OMR	XXX LY	XXX OMR	XXX LY	XXX OMR /LY
Comparator health technology	XXX OMR	XXX LY			
**Health benefit expressed in QALYs**					
Investigated health technology	XXX OMR	XXX QALY	XXX OMR	XXX QALY	XXX OMR /QALY
Comparator health technology	XXX OMR	XXX QALY			

Abbreviations: ICER Incremental Cost-Effectiveness Ratio, OMR Omani rial, LY life years, QALY Quality-Adjusted Life Year.

Sensitivity and scenario analyses are key for evaluating the reliability of cost-effectiveness results. Deterministic sensitivity analysis (DSA) is mandatory, involving changes to individual inputs to assess their impact, with results shown in Tornado diagrams. Probabilistic sensitivity analysis (PSA) is optional, using probability distributions and multiple simulations to capture uncertainty, presented via cloud diagrams and acceptability curves. Scenario analysis explores the effects of altering key assumptions or model settings, enhancing the robustness of the evaluation.

#### Budget impact analysis (BIA)

BIA is a key component of HTA methodology guidelines, focusing on the affordability of the investigated technology. Patient numbers and health care costs borne by the health care payer over four years without discounting should be taken into account in BIA. Assumptions in BIA and cost-effectiveness analyses should be aligned and rely on Oman-specific resource use and cost data. BIA must transparently present input sources, consider gradual patient inclusion, and account for healthcare payer costs only. Sensitivity analysis, particularly deterministic, is mandatory to assess uncertainties. Patient population estimates should include disease incidence, prevalence, subgroups, and market share. Results must specify annual and cumulative direct medical costs for the overall targeted population (and whenever possible for subgroups), emphasising the net budget impact.

#### Other aspects (ethical, organisational, patient, and social)

The HTA methodology guidelines extend beyond assessing the clinical and cost-effectiveness of technology by also including evidence on patient, caregiver, and organisational perspectives. Omani HTA guidelines recommend increasing patient involvement in the HTA process by encouraging patients to share their voices, providing clear information to help them make choices, and protecting their privacy. They also emphasise paying special attention to vulnerable groups, overcoming access barriers across different social and cultural contexts, and ensuring transparency regarding conflicts of interest. Additionally, organisational considerations specify how new health technologies can be effectively integrated into the healthcare system. This includes their impact on patient pathways and service delivery, the need for staff training or workflow adjustments, the availability of human resources and infrastructure, additional costs such as equipment or quality assurance, and management or cultural factors that may affect acceptance.

#### Transparency

The participants agreed that the publication of the HTA dossier is important for transparency to inform clinicians, patients and researchers in order to enhance accountability and public trust. This is achieved by preparing two versions of the HTA dossier, ‘Full Version’ for experts and decision-makers, which will include critical appraisal, pricing and reimbursement, containing comprehensive data. While the ‘Published Version’ for the general public containing information such as epidemiology, unmet needs, efficacy, safety and aggregated health gain of the investigated technology, current treatment mix, resource use and treatment costs of patients and protect the commercially sensitive details, especially the proposed price of the new technology. Full details of the HTA dossier to be published are found in additional file 1 (Supplemental Table S1).

Full disclosure of conflicts of interest is mandatory in HTA submissions, including the roles and compensation of contracted organisations and experts who were involved in the preparation of the HTA dossier.

A summary for the key features in the HTA guidelines is presented in Table 3.

**Table 3.** Summary of key features in the HTA guidelines File name: Additional file 1 File format:.pdf Title of data: Transparency of Oman HTA guidelines.

Principle	Recommendations
Target Indication	The target indication for reimbursement may be more specific but not broader than the approved indication. The population in the HTA dossier cannot differ from the targeted patient population in the reimbursement application
Disease Area	Clearly describe the disease epidemiology, current patient management and pathways, the unmet medical needs and an overview of currently reimbursed technologies in the target indication.
Comparator	The comparator must be authorised, reimbursed, scientifically supported, widely used technology. Any deviation from standard technologies should be justified.
Perspective	Mandatory: Health care perspective. Optional: Societal perspective.
Type of economic evaluation	CUA for any technologies with health gain, expressed in incremental QALYs. Cost-minimization analysis for technologies with no health gain to the comparator.
Time horizon	The time horizon must be long enough to capture the full cost and outcome implications of the health technology.
Modelling	Use models like decision trees or Markov models for long-term evaluation, justifying your choice and ensuring transparency in inputs and assumptions.
Discounting	3% discount rate apply to both costs and outcomes to reflect the time preference for health gains and costs occurring in the future.
Uncertainty analysis	Mandatory deterministic sensitivity analysis must vary key inputs by ±10%, while probabilistic sensitivity and scenario analyses are recommended for robustness.
CET	Baseline CET is 1x GDP per capita, with modifiers for significant health gains (max 3x), health policy priorities (2x) and technologies in rare diseases (2x).
Presenting results	Presented results should include health benefits and costs for the technology and comparator. ICER must be presented in a reproducible format.
Budget Impact Analysis	4-year time horizon should be applied by considering direct medical costs related to the gradual uptake of the technology. Costs should be aligned with local practices and pricing structures.
Transparency	Full disclosure of conflicts of interest, expert contributions, and a public version of HTA dossiers are mandatory to enhance transparency.

Abbreviations: HTA Health Technology Assessment, CUA Cost Utility Analysis, QALYs Quality Adjusted Life Year, CET Cost Effectiveness Threshold, GDP Gross Domestic Product.

## Discussion

The development of clear and transparent HTA guidelines is one of the key components required to establish an effective HTA system (World Health Organization, 2015). These processes are a bridge between HTA doers (including companies, HTA consultants and HTA bodies) and users (decision-makers). Regarding patient involvement, the guidelines recommend that patients actively share their perspectives and take part in the assessment process.

This paper provides a summary of the Omani HTA methodological guidelines that must be followed when conducting and reporting of reimbursement submission for new health technologies in Oman. Primarily, it is important for HTA doers to ensure the quality and standards of technology assessment process.

These guidelines are developed based on the existing best practices and recommendations from multiple stakeholders. Adopting local guidelines tailored to unique healthcare challenges and priorities will encourage transparency and facilitate the use of local data (Al Rashdi et al., 2024; Kaló et al., 2013). This will ultimately support sustainable healthcare financing, improve health outcomes and ensure equitable access to high-quality care across the country (World Health Assembly, 2014). This guideline contains six sections related to the following: target indications, medical assessment, economic evaluation, budget impact analysis, and other aspects ethical, organisational, patient, and social aspects. These sections create a comprehensive guideline by touching on all aspects necessary to conduct a health technology evaluation.

Finally, the HTA guidelines have been developed based on current best practices. However, they will be reviewed and updated every three years to stay aligned with evolving standards and best practices in HTA.

Our approach to developing the guidelines is broadly aligned with the good practice recommendations outlined by Botwright et al. (2024), which emphasise the importance of setting clear objectives, engaging diverse stakeholders, establishing strong institutional arrangements, and regularly monitoring the success of HTA guidelines (Botwright et al., 2024).

The principle of diverse stakeholder engagement is reflected in our multistakeholder workshop, which brought together regulators, payers, policymakers, and healthcare providers. Additionally, in line with Botwright et al.’s recommendation for ongoing monitoring, our guidelines will be reviewed and updated every three years to ensure their continued relevance and effectiveness (Botwright et al., 2024).

When comparing our guidelines with others in the region, the UAE recently published HTA guidelines through the Department of Health in Abu Dhabi (Department of Health, 2025). Both our guidelines and that of the UAE emphasised that the target indication and population must be explicitly defined in submissions, and that the target indication cannot extend beyond the approved or registered indication. In both guidelines, QALYs were preferred as the primary outcome measure, and the time horizon for cost-effectiveness analyses should be sufficiently long to capture all relevant health outcomes (Department of Health, 2025). A difference, however, is observed in the budget impact analysis time horizon: Oman recommends four years, whereas the UAE specifies three years (Department of Health, 2025).

Looking at countries with comparable economic contexts (high income countries), most guidelines recommend conducting submissions from the payer perspective, with the societal perspective considered optional, as seen in Croatia, and Hungary (Agency for Quality and Accreditation in Health Care, 2011; Emberi Erőforrások Minisztériuma, 2024). While many countries do not specify whether HTA reports are publicly available, Oman explicitly states that HTA reports will be published and made transparent, similar to practices in a limited number of countries such as Croatia, England, Scotland, and Norway (Agency for Quality and Accreditation in Health Care, 2011; National Institute for Health and Care Excellence, 2022; Norwegian Medicines Agency, 2012; Scottish Medical Consortium, 2022).

Nevertheless, there are limitations regarding the 3% discount rate justified as per existing guidelines and participants voting in the survey, since there is no official discount rate for the health effect in Oman. There is a possibility to conduct future research to calculate the discount rate for cost and health effects in the Omani healthcare sector. Additionally, no participants from the private health sector or patient representatives were involved in the preparation of this guideline. While we acknowledge the value of patient and patient representative involvement, the development of such groups in Oman remains in its early stages. Therefore, patients were not included in the development of the guidelines; however, the guideline encourages patients to contribute their perspectives during the assessment phase of submissions, to help inform and improve future decision-making.

## Conclusion

Acceptance of Omani HTA methodological guidelines by the Ministry of Health represent an important milestone in strengthening the evidence base for policy decisions in Oman. These new guidelines offer a structured evidence-based approach for the value judgment of health technologies. To support the production of high-quality and more consistent evaluations, the guidelines emphasise transparency and the use of local data. Standardising HTA processes not only improves decision-making but also allows for more effective prioritisation of healthcare investments, leading to better resource allocation and improved health outcomes.

## Supplementary Material

Additional File 1.docx

## Data Availability

The authors confirm that the data supporting the findings of this study are available within the article.

## Generative artificial intelligence

CHATGPT was used to edit limited sentences and not in the creation or generation of the manuscript. After using this tool/service, the authors reviewed and edited the content as needed and take full responsibility for the content of the publication.

## Description of data

The document outlines the components of the HTA dossier, specifying which elements are mandatory for publication, which are recommended, and which are not recommended, presented in a table format.
