# Supplementary material for: Methodological guidelines for Health Technology Assessment in Oman
Source: J Pharm Policy Pract. 2025 Dec 9;18(1):2596523. doi: 10.1080/20523211.2025.2596523 (PMC12690758; doi:10.1080/20523211.2025.2596523)
Supplement: Additional File 1.docx [file JPPP_A_2596523_SM0734.docx]

Transparency of Oman HTA guidelines

Table S1: Details of the HTA dossier to be published

| **HTA dossier chapters** | **Publication of details** |
| --- | --- |
| Epidemiology of the target indication (incidence, prevalence) | Mandatory |
| Current patient pathways with highlights on unmet medical need | Mandatory |
| Efficacy and safety of the new technology | Mandatory |
| Methodology of calculating health gain by the new technology | Mandatory |
| Estimated health gain | Mandatory |
| Methodology of cost calculations | Mandatory |
| Estimated current resource use and treatment costs of patients | Mandatory |
| Proposed price of the new technology | No publication |
| Estimated resource use and treatment costs of patients with the new technology | No publication |
| Economic modelling methodology (model type, time horizon, discount rate, etc.) | Mandatory |
| Cost-effectiveness results (incr. health gain, costs and cost-effectiveness ratio) | Recommended |
| Sensitivity analysis results for the cost-effectiveness analysis | Recommended |
| Methodology of budget impact calculations | Mandatory |
| Current treatment mix of patients | Mandatory |
| Estimated patient numbers and market share of new technology in the next 3-5 years | Recommended |
| Budget impact of the new technology | Recommended |
